# Supplementary material for: A modelled analysis of the impact of COVID-19-related disruptions to HPV vaccination
Source: eLife. 2023 Oct 13;12:e85720. doi: 10.7554/eLife.85720 (PMC10575627; doi:10.7554/eLife.85720)
Supplement: Supplementary file 1. — (A) Age-specific cancer rates for females in Australia (per 100,000), 2020 projections; (B) age-specific cancer rates for males in Australia (per 100,000), 2020 projections; (C) human papillomavirus HPV attributable fractions and HPV9 preventable proportions for the cancers modelled; (D) estimated number of cervical and total cancer cases in modelled scenarios for explicit screening (main analysis) vs incidence-based approach. [file elife-85720-supp1.docx]

**Supplementary File 1A**

Age-specific cancer rates for females in Australia (per 100,000) – 2020 projections^1^

| **Age** | **Anal** | **Oropharyngeal** | **Vaginal** | **Vulvar** |
| --- | --- | --- | --- | --- |
| 00–04 | 0 | 0 | 0.1 | 0.1 |
| 05–09 | 0 | 0.1 | 0.0 | 0.0 |
| 10–14 | 0 | 0 | 0.0 | 0.0 |
| 15–19 | 0.1 | 0 | 0.0 | 0.1 |
| 20–24 | 0 | 0 | 0.0 | 0.1 |
| 25–29 | 0.1 | 0.1 | 0.2 | 0.3 |
| 30–34 | 0.2 | 0.3 | 0.0 | 0.0 |
| 35–39 | 0.9 | 0.1 | 0.0 | 0.9 |
| 40–44 | 0.9 | 0.4 | 0.7 | 1.1 |
| 45–49 | 2.2 | 1.3 | 0.5 | 2.6 |
| 50–54 | 3.3 | 2.1 | 1.4 | 4.3 |
| 55–59 | 7.1 | 3.7 | 1.1 | 4.6 |
| 60–64 | 7 | 4.5 | 1.2 | 7.5 |
| 65–69 | 7.6 | 3.1 | 2.5 | 8.4 |
| 70–74 | 10 | 2.7 | 1.8 | 12.0 |
| 75–79 | 9 | 1 | 1.8 | 11.3 |
| 80–84 | 7.3 | 2.4 | 1.4 | 16.4 |
| 85–89 | 5.5 | 1.1 | 4.9 | 24.5 |
| 90+ | 8.2 | 3 | 7.5 | 14.2 |

**Supplementary File 1B**

Age-specific cancer rates for males in Australia (per 100,000) – 2020 projections^1^

| **Age** | **Anal** | **Oropharynx** | **Penile** |
| --- | --- | --- | --- |
| 00–04 | 0.0 | 0 | 0.0 |
| 05–09 | 0.0 | 0 | 0.0 |
| 10–14 | 0.0 | 0 | 0.0 |
| 15–19 | 0.0 | 0 | 0.0 |
| 20–24 | 0.0 | 0.1 | 0.0 |
| 25–29 | 0.1 | 0 | 0.0 |
| 30–34 | 0.0 | 0.1 | 0.2 |
| 35–39 | 0.1 | 0.2 | 0.3 |
| 40–44 | 0.0 | 3.3 | 0.4 |
| 45–49 | 2.2 | 7.4 | 0.8 |
| 50–54 | 2.9 | 11.7 | 1.4 |
| 55–59 | 3.8 | 22.1 | 1.7 |
| 60–64 | 6.6 | 20.4 | 2.0 |
| 65–69 | 5.3 | 18.3 | 1.0 |
| 70–74 | 6.5 | 12.9 | 3.9 |
| 75–79 | 7.4 | 10.1 | 5.4 |
| 80–84 | 5.5 | 8.4 | 8.4 |
| 85–89 | 7.7 | 3.9 | 7.0 |
| 90+ | 8.5 | 4.3 | 8.5 |

**Supplementary File 1C**

HPV attributable fractions and HPV9 preventable proportions for the cancers modelled

| **Cancer** | **% HPV attributable** | **% of HPV-attributable preventable by HPV9** | **Overall HPV9-preventable** | **Population** | **Source** |
| --- | --- | --- | --- | --- | --- |
| Anal | 88.3% | 95.9% | 84.7% | International | de Martel et al, 2017^2^ |
| Cervical | 100% | 93.0% | 93.0% | Australia | Brotherton et al, 2017^3^ |
| Oropharyneal/ tonsillar | 63.5% | 89.7% | 57.0% | Australia | Hong et al, 2016^4^; Castellsague et al, 2016^5^ |
| Penile | 50.0% | 84.6% | 42.3% | International | de Martel et al, 2017^2^ |
| Vaginal | 78.0% | 85.3% | 66.5% | International | de Martel IJC 2017^2^ |
| Vulvar | 24.9% | 87.1% | 21.7% | International | de Martel IJC 2017^2^ |

HPV9 = 9-valent HPV vaccine, protecting against HPV 6/11/16/18/31/33/45/52/58

**Supplementary File 1D**

Estimated number of cervical and total cancer cases in modelled scenarios for explicit screening (main analysis) vs incidence-based approach*. (Values in parentheses are additional cases compared to ‘no disruption’ scenario)

| **Modelled scenarios** | **Cervical (explicit screening)** | **Cervical (incidence-based)** | **Total females (explicit screening)** | **Total females (incidence- based)** | **Total females and males (explicit screening)** | **Total females and males (incidence- based)** |
| --- | --- | --- | --- | --- | --- | --- |
| **2008 cohort** |  |  |  |  |  |  |
| Unvaxed | 788 | 980 | 2,353 | 2,545 | 3,923 | 4,115 |
| No disruption | 62 | 125 | 889 | 952 | 1,532 | 1,596 |
| Scenario 1 | 63  (1) | 127  (2) | 892  (3) | 955  (3) | 1,537  (4) | 1,600  (4) |
| Scenario 2 | 74  (12) | 158  (33) | 928  (39) | 1,012  (60) | 1,603  (70) | 1,686  (90) |
| Scenario 3 | 250  (188) | 430  (305) | 1,334  (445) | 1,515  (563) | 2,282  (750) | 2,463  (867) |
| **2008 and 2009 cohorts** | |  |  |  |  |  |
| Unvaxed | 1,576 | 1,960 | 4,706 | 5,090 | 7,846 | 8,231 |
| No disruption | 125 | 248 | 1,777 | 1,901 | 3,061 | 3,185 |
| Scenario 4 | 588  (463) | 1,024 (776) | 2,895 (1,118) | 6,034 (4,133) | 4,954  (1,892) | 5,390  (2,205) |

All values, including differences between scenarios, are rounded to whole numbers of cases.

Unvaxed: assuming no HPV vaccination in cohort (s), no disruption: HPV vaccination in males and females at age 12 with coverage of 82.4% in females and 75.5% in males; scenario 1: 1-year delay in vaccination catch-up, scenario 2: slow catch-up: 1 to 7-year delay; scenario 3: no catch-up (herd effects only 2008 cohort); scenario 4: no catch-up (herd effects only; 2008 and 2009 cohorts.

* ie using the same methodology as employed for non-cervical HPV-related cancers.

**References**

1. Australian Institute of Health and Welfare (AIHW) 2020 Cancer Data in Australia; Canberra: AIHW. https://www.aihw.gov.au/reports/cancer/cancer-data-in-australia/
2. De Martel C, Plummer M, Vignat J, Franceschi S. Worldwide burden of cancer attributable to HPV by site, country and HPV type. International journal of cancer. 2017;141(4):664-70.
3. Brotherton JM, Tabrizi SN, Phillips S, Pyman J, Cornall AM, Lambie N, Anderson L, Cummings M, Payton D, Scurry JP, Newman M. Looking beyond human papillomavirus (HPV) genotype 16 and 18: defining HPV genotype distribution in cervical cancers in Australia prior to vaccination. International journal of cancer. 2017;141(8):1576-84.
4. Hong A, Lee CS, Jones D, Veillard AS, Zhang M, Zhang X, Smee R, Corry J, Porceddu S, Milross C, Elliott M. Rising prevalence of human papillomavirus–related oropharyngeal cancer in Australia over the last 2 decades. Head & neck. 2016;38(5):743-50.
5. Castellsagué X, Alemany L, Quer M, Halec G, Quirós B, Tous S, Clavero O, Alòs L, Biegner T, Szafarowski T, Alejo M. HPV involvement in head and neck cancers: comprehensive assessment of biomarkers in 3680 patients. Journal of the National Cancer Institute. 2016 Jun 1;108(6):djv403.
